# The ban on blood donation on men who have sex with men: time to rethink and reassess an outdated policy

**DOI:** 10.11604/pamj.2017.27.99.12891

**Published:** 2017-06-08

**Authors:** Georgios Karamitros, Nikolaos Kitsos, Ioanna Karamitrou

**Affiliations:** 1 Medical School, Aristotle University of Thessaloniki, Thessaloniki, Greece; 2 Faculty of Medicine, University of Southampton, Southampton, United Kingdom; 3 Law School, Democritus University of Thrace (DUTH), Komotini, Greece

**Keywords:** HIV, AIDS, donation, blood banks, homosexuality, risk assessment

## Abstract

During the 1980s the HIV/AIDS epidemic outbreak occurred. Due to the high prevalence of the disease on men who had sex with men (MSM) a lifetime ban on blood donations on men who had sex with men (MSM) was implemented. In the recent years, organizations like the European Union (EU) and the World Health Organization (WHO) have established new guidelines introducing the term of “risky sexual behavior” without any reference to the sex orientation of the potential donor, however many countries are hesitant to review the ban on men who had sex with men (MSM). Given the lack of screening methods for HIV back in the '80s the ban on men who had sex with men seemed like the only choice in order to limit the disease. However, nowadays the screening methods have advanced and the possibility of a transfusion related HIV infection is extremely low. Many countries, considering the new data available, have reformed their policies and moved from the lifetime ban to 5-year and 1-year deferrals but only a fraction of countries have adopted the guidelines for the “risky sexual behavior” assessment. The ban that forbid men who have sex with men from donating blood was implemented more than 30 years ago. During the '80s, the epidemiology was different and it seems not only hypocritical but also naïve to rely on guidelines that are far outdated and old-fashioned. The medical community has a duty to secure safe blood for every person who might need it, let us not waste safe potential donors and stigmatize them by focusing on outdated policies.

## Opinion

Just over 100 million blood donations are collected all over the world annually [1]. In order to protect blood product recipients and avoid transfusion-related infections the World Health Organization (WHO) and the European Union (EU) as well as Health Ministries of different countries around the world have implemented exclusion criteria for potential future blood donors [2, 3]. The European Union and the World Health Organization exclude from donating blood 'a person whose sexual behavior puts them at high risk of acquiring severe infectious diseases that can be transmitted by blood'. However even though the guidelines provided by these organizations are impartial towards the sexual orientation of the potential donor, many countries around the world-such as Austria, Belgium, Denmark, Estonia, Germany, Israel and Norway-continue to ban all men who have sex with men (MSM) from contributing via blood donation [4] and strictly adhere to the permanent deferral that the international regulatory authorities introduced in 1977.

**The historical and social context surrounding HIV/AIDS:** In early 1983, there were indisputable evidence suggesting that the cause of Acquired Immune Deficiency Syndrome (AIDS) was a bloodborne pathogen and more specifically a virus that could infect people through blood and sex. It was evident therefore; that there was a constant fear that blood banks around the world might be contaminated by an unidentified and possibly lethal pathogen [5]. It became clear afterwards, that between 1970 and 1980 20.000 Human Immunodeficiency Virus (HIV) infections and 200.000 Hepatitis C Virus (HCV) infections had been attributed to the contaminated blood that had been provided to blood products recipients [6]. Around the same period in Canada, there were 1.200 HIV infections and 25.000 HCV infections due to contaminated blood [7]. However, there were not any significantly trustworthy screening methods available at this point. Meanwhile, the public opinion was alarmingly concerned about this unknown disease. Infectious disease specialists had been overseeing the sudden outbreak of the disease since 1981, when great numbers of until lately healthy male individuals began dying of pneumonia caused by Pneumocystis jiroveci. Most of them had recently been diagnosed with Kaposi sarcoma, a rare malignancy, which is the “trademark” of this new immune system-ravaging disease. As years went by, AIDS seemed to have a particular affinity towards homosexual men and people who were drug addicts and performed drug injections. With the numbers of HIV infection being constantly on the rise and given the higher prevalence of HIV infections among MSM compared to the general population, a ban was adopted by blood banks in 1985. The ban, also called “deferral”, prohibited high-risk groups, like MSM to donate blood [5]. This kind of action had been adopted not only in the USA, but also by the late 1980s almost all of North America and Europe had formed similar strategies to counteract the silent threat [8].

**Evidence supporting MSM blood donation policy change:** Our attempt to accurately evaluate the result of the reassessment of the ban of MSM on blood donation guidelines comes across certain challenges: most blood services in different countries do not publish HIV surveillance data and most accessible studies on this topic are low quality observational studies [9]. To confront these challenges we relied on epidemiological studies, the accuracy of HIV screening technology and studies based on mathematical models. At the time that the ban on MSM had been enforced, no HIV screening test existed. Since 1983, HIV screening tools have significantly improved and the improvements can be categorized into two key elements. Firstly, the window period, which is the time that a person who is infected with the virus before it is detectable by a test, has been shortened. Secondly, the test's sensitivity had been improved, which means that now the tests can trustworthily identify an infected person after the window period has passed. In 1987, the HIV testing methods had a window period of 6 to 14 weeks. With an 8-week window period it has been estimated that with the HIV testing methods of 1987 there was a chance of HIV infection in every 153.123 units of blood [10]. However, policies of blood banks nowadays have in their arsenal advanced serologic screening technologies which approach 100 percent in sensitivity and specificity [11]. Current HIV testing technologies reduce the risk of HIV-contaminated blood infection to 1 per 8-to-12 million donations [11] (Table 1). Additionally, according to The Joint United Nations Programme on HIV/AIDS (UNAIDS) [12] the distribution of new HIV infections among adults altered in the past years. In 2015 only 27% of the new HIV infections were attributed to the MSM population, while the “rest of the population” group accounted for 39% of the new HIV infections [12]. Taking these new data into consideration, we can unsderstand that the balance has shifted and homosexual people or MSM in general, are not the population group that is mainly affected by HIV these days.

**Table 1:** HIV screening methods available

Test	Sample Type	Sensitivity(percent)	Specificity(percent)
OraQuick advance Rapid HIV1/2 Antibody test	Oral fluid	99.3	99.8
	Whole blood	99.6	100
	Plasma	99.6	99.9
Clearview complete HIV1/2	Whole blood	99.7	99.9
	Serum and plasma	99.7	99.9
Clearview HIV1/2 STATPAK	Whole blood	99.7	99.9
	Serum and plasma	99.7	99.9
Reveal G-3 rapid HIV-1 antibody test	Serum	99.8	99.1
	Plasma	99.8	98.6
Uni-gold recombingen HIV	Whole blood	100	99.7
	Serum and plasma	100	99.8
Multispot HIV-1/HIV-2 rapid test	Serum	100	99.9
	Plasma	100	99.9
INSTI HIV-antibody test	Plasma	99.9	100
	Whole blood (venipuncture)	99.9	100
	Whole blood (finger stick)	99.8	99.5

**Reassessing the ban on MSM:** Given the above-mentioned data, the American red cross and the American association of blood banks have come to the conclusion that the policy that bans MSM to donate blood is outdated and there is no medical or other scientific reason to support it [13]. Since countries had gradually lifted the ban on MSM and implemented a 1-year deferral period, it became possible to evaluate the direct result of these policy changes. England, Scotland and Wales implemented the 1-year deferral policy for MSM in 2011. A continues study found that with the 1-year deferral policy an extra 46 percent of MSM were eligible to donate blood compared to a 5-year deferral policy [14], while the risk of HIV-contaminated blood infection increased “only marginally” with the 1-year deferral compared to lifetime ban, resulting in one HIV-contaminated blood donation every 455 years [15]. Although 1-year deferral guidelines have been implemented in many countries around the globe like the United States, Japan, Brazil, the United Kingdom and Australia [4], and there are evidence-based models that strongly support the 1-year deferral period there are countries in Europe like Spain and Italy which have implemented individual assessment of potential blood donors that seem rather promising [16]. In Spain and Italy there is an individual approach of the potential donor and there is an evaluation of MSM into “low risk” and “high risk” for HIV. For example, a MSM is considered “low risk” for HIV if he is in a long-term monogamous relationship and therefore, he is a “safer” candidate for blood donation. On the other hand, heterosexual men who had multiple sexual partners and unprotected sex during the last month, is considered a “high risk” candidate. This individual assessment policy seems less discriminatory and can assess potential donors regardless of their sexual orientation and strictly based on what is described as “risky sexual behavior”.

## Conclusion

The lesbian, bisexual, transgender/two-spirited and queer (LGBTQ+) community has a history of discrimination and has often been dealt with racism from the healthcare system [17]. It is not difficult to understand that with current policies the society drives the LGBTQ+ people not only to hide their sexual orientation but also to distrust the medical care system that supposedly take care of them [18]. The past three decades of strenuous activism have produced important gains for the LGBTQ+ rights. For example, nowadays homosexuality is far more approved worldwide than back in 1987. Alteration in the public notion has resulted into legislative gains for LGBTQ+ rights. These include the Supreme's Court ruling, on 26^th^ of June 2015, that same-sex couples can marry nationwide in the United States of America. Additionally to growing social support, an alarmingly high number of scientists, medical personnel and blood-banking organizations, such as the Red Cross, WHO and the EU have taken steps to question the suitability of the ban on MSM blood donation. However, even with the guidance of those worldwide known organizations still many countries are extremely hesitant to review the MSM blood donation policy (for numerous reasons) and continue the vicious circle of discrimination and racism (Table 2). MSM banned from donating blood almost 30 years ago when it was considered of crucial importance for the safekeeping of public health. Back in the day, the epidemiology was different and it seems not only hypocritical but also naïve to rely on guidelines that are far outdated and old-fashioned. And even through implementing a 1-year deferral seems a step in the right direction, it seems like a rather crippled step. Following the guidelines of respected organizations like WHO which speak about risk-based deferrals and “risky-sexual behaviors” seems like a dire need. We have a duty to secure safe blood for every person who might need it, let us not waste safe potential donors and stigmatize them by focusing on outdated policies.

**Table 2:** Arguments “For” and “Against” the reassessment of the ban on men who have sex with men (MSM)

Arguments “For”	Arguments “against”	
Changes in nowadays epidemiology shows decreased incidence of HIV in MSM	Increased risk of acquiring a blood born pathogen among blood products recipients	
Increased need for blood donors	Potential of new emerging pathogens	
Successful results from the implementation of the “individual risk assessment” policy	Difficulties to implement the “individual risk assessment” policy	
Current policies are discriminatory and stigmatizing towards a certain population group		

## Competing interests

The authors declare no competing interest.
